# Towards an Embodied Signature of Improvisation Skills

**DOI:** 10.3389/fpsyg.2019.02441

**Published:** 2019-11-01

**Authors:** Alexandre Coste, Benoît G. Bardy, Ludovic Marin

**Affiliations:** EuroMov, Univ. Montpellier, Montpellier, France

**Keywords:** improvisation, creativity, expertise, motor signature, embodied cognition

## Abstract

Improvisation is not limited to the performing arts, but is extended to everyday life situations such as conversations and decision-making. Due to their ubiquitous nature, improvisation skills have received increasing attention from researchers over the last decade. A core challenge is to grasp the complex creative processes involved in improvisation performance. To date, many studies have attempted to provide insight on brain activity and perceptual experiences when perceiving a performance, especially in musical or artistic form. However, watching/listening a performance is quite different than acting in a performance or performing daily-life activities. In this Perspective, we discuss how researchers have often missed key points concerning the study of improvisation skills, especially by ignoring the central role of bodily experiences in their formation. Furthermore, we consider how the study of (neglected) motor component of improvisation performance can provide valuable insights into the underlying nature of creative processes involved in improvisation skills and their acquisition. Finally, we propose a roadmap for studying improvisation from the acquisition of kinematic data in an ecological context to analysis, including the consideration of the coalition of (individual, environmental and task) constraints in the emergence of improvised behaviors.

## Introduction

In psychology, improvisation is typically conceived as a creative process without a script or anticipated preparation (Gueugnon et al., 2016a, b). The idea that improvisers spontaneously create a novel product *ex nihilo* rather than from a pre-existing material or substrate has a powerful intuitive appeal. Yet, this idea is not well supported by empirical evidence (Brown, 2000). Instead, improvised products that are central in many fields, including performing arts (e.g., music, theater or dance) and everyday life (e.g., improvising a dinner, a speech or actions in sport settings), are conceived to be shaped by the lifetime history of individuals, especially via their past (bodily) experiences and training (Chelariu et al., 2002). According to this perspective, each element is pre-existing, but the way improvisers can combine them is a unique creation of the present moment. Improvisers must therefore master a whole package of elements (i.e., constituting their repertoire) from which they gradually learn to build their own improvised composition (Azzara and Grunow, 2010). In this respect, improvisation expertise requires a large amount of practice and experience, leading to a myriad of perceptual, cognitive and motor changes over time (e.g., Ericsson et al., 1993; Pinho et al., 2014). Although expertise has long been at the heart of research in psychology, sports sciences and a wide range of other fields, studies dealing with improvisation expertise are paradoxically limited, and the underlying mechanisms of learning across the various stages of improvisation skills construction are largely unclear. A potential reason that limits our knowledge of improvisation skills could be the difficulty to capture the “higher-order cognitive processes” underlying improvisation performance in standardized laboratory tasks. To date, many studies have attempted to provide insight on brain activity and perceptual experiences when perceiving a performance, especially in musical or artistic form. Thinking from a broader perspective, we consider in this article that previous experimental investigative contexts are too limited such that the results may not extend to more complex realistic situations. In addition, we show that some key ideas from different disciplines have been overlooked in improvisation skills literature, such as the consideration of motor coordination in collective improvisation. Moreover, we believe that creativity can be more rigorously quantified by studying body movements in order to measure how people recycle motion (improvise) over time and determine whether the movement combination was creative (i.e., statistically rare) or not.

### Improvisation and Creativity in the Arts

Improvisation is traditionally viewed as the essence of performing arts, when artists are devoted to an act of creation “on the spot” within a well-defined framework. It is therefore not surprising that the greatest scientific literature on improvisation can be found among the arts, namely music and dance (Pressing, 1984). In particular, much of our knowledge about improvisation comes from neuroimaging studies that shed light onto which brain regions are involved during dance/music perception (Calvo-Merino et al., 2004; Engel and Keller, 2011) or improvised music production (Bengtsson et al., 2007; Berkowitz and Ansari, 2008; Limb and Braun, 2008; Pinho et al., 2014). It is now well documented that improvisation is most commonly associated with activation of the premotor and prefrontal cortex areas (Pinho et al., 2014; Beaty, 2015), two cortical areas known to play a significant role in planning/initiation of voluntary motor movements (e.g., Lu et al., 2012) and creativity (e.g., Dietrich, 2004), respectively. Creativity, i.e., the capacity to generate both novel and meaningful events, constitutes thus a key element of improvised products. Although it is hard to differentiate creativity from improvisation since they are closely nested, what characterizes improvisation performance relies above all on its spontaneous character, its aesthetic values of perfection/imperfection and does not imply necessarily a radical novelty. In addition, improvisation has an intrinsic motor component that is not necessarily found in all domains of creativity (e.g., generating creative ideas) except for the specific case of motor creativity (Orth et al., 2017). In this context, we focus in this article on the motor aspect of improvisation performance, especially because what happens “outside the head” during performance remains largely under-explored in the literature. This is paradoxical when one considers that “*the primary instrument through which improvisation takes place is the human body and its interactions with other bodies*” (Carter, 2000, p. 182).

### Improvisation in Real-Life and Skill Transfer

Little is known about improvisation outside the arts; however, improvisation skills occupy a central place in a broad landscape, from arts to sports to everyday activities. In sports such as football, basketball and tennis, the scenario of the match is unpredictable and players must constantly adapt their plans/actions to the current situation while respecting the framework defined by the rules of the game. Similarly, improvisational skills are crucial in situations where verbal and/or non-verbal communication can be challenging, such as communicating in a foreign language. It then appears necessary to extend the study of improvisation skills and their acquisition to a variety of tasks. The study of improvisational skills in different situations would help to uncover whether and to what extent skills are transferrable across tasks, domains, and/or disciplines. For example, are dancers’ improvisational skills specific to the dance of which they are experts, or can they also be transferred to music or daily social situations?

### Challenges of Measuring Improvisation

The quantification of improvisation can be challenging, from a neuroscience approach (e.g., using fMRI or EEG), because the use of novel tasks involving substantial body movements often generate measurement artifacts (Pressing, 1984). Therefore, there is motivation for alternative ways to quantify motor improvisation. The proposed approach therein overcomes this challenge by directly analyzing the flow of information conveyed by human movements in order to gain insights into the creative processes underpinning improvisation performance. This would be a viable strategy, as we strongly believe that improvisation skills are embodied in our action-perception synergies, such that our lifetime bodily experiences influence our improvisation skills, and in turn, our improvisation skills influence our body movements.

## A New Approach to Improvisation Grounded on Motor Component

Watching outstanding individuals’ performance is simply captivating and attests to the fact that there is something special in the way they move. Moving bodies both receive and transmit a wealth of information about a person, including intentions, emotions, personality or identity that we can effortlessly perceive (e.g., Troje, 2002). There are good reasons to believe that improvisational skills, similarly to emotions or intentions, color movements. For example, it has been empirically demonstrated that movement qualities of expert improvisers differ from those of novices (Noy et al., 2011; Issartel et al., 2017). Using the *mirror game* (Noy et al., 2011), a simple yet effective paradigm for studying two people improvising hand movements with different social roles (leader/follower/no designated leader), Noy et al. (2011) found that experts create more complex (i.e., creative) and synchronized motion than novices when there is no designated leader. In another behavioral study, Issartel et al. (2017) compared motion characteristics in the *mirror game* of three groups with distinct levels of expertise in improvisation dance (novice, intermediate, expert). Results revealed that each group had a very specific movement organization and that motor creativity increased with expertise. Thereafter, a series of studies attempted to provide insights into how these improvisation skills evolve over time from practice. For instance, Gueugnon et al. (2016b) showed that movement richness of novice pairs in the *mirror game* increased across time, but solely for dyads performing unintended synchronized movements. Taken together, these results suggest (i) the existence of a motor signature of improvisation expertise and (ii) improvisation skills can be enhanced through practice—as the old adage goes, “practice makes perfect.” Thus, similar to athletes who rigorously follow an intensive training to perfect their technical, tactical and physical skills for performance purposes, improvisers must spend a large number of hours of practice devoted to skill-building. It is important to note however, that, except the work of Issartel et al. (2017), who conjointly studied both solo-improvisation and joint-improvisation to establish the influence of the social interaction, other studies related to the *mirror game* have addressed mainly joint coordination. Solo and joint improvisation differ in that joint improvisation incorporates (in addition to solo skills) dyadic coordination. This difference may have major consequences on improvised behaviors. For instance, Issartel et al. (2017) reported that movement richness in joint improvisation, where participants were explicitly asked to be coordinated, was significantly reduced compared to the solo condition irrespective of the level of expertise (novice, intermediate, expert). This finding suggests that both the (social) environment and the goal of the task (collaborative task) interact and play a crucial role in shaping improvisation behaviors.

## The Role of Constraints in the Emergence of Improvised Behaviors

**FIGURE 1:**
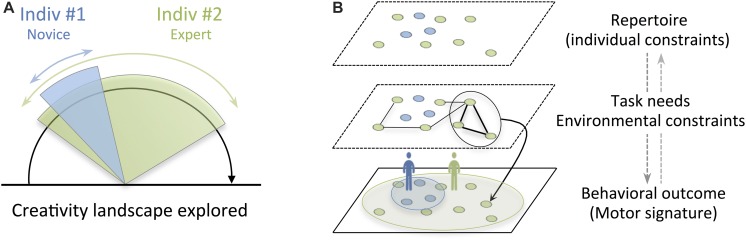
**(A)** Schematic depiction of the motor creativity of two individuals with different levels of expertise in improvisation. The landscape of creativity explored by the expert (indiv#2) is supposed to be broader than that of the novice (indiv#1). **(B)** Illustration of the three layers leading to the emergence of improvised behaviors. The first layer corresponds to the individual repertoire formed largely by past body activity experiences. The second layer shows how behavioral solutions emerge through the exploration of the individual repertoire under external constraints (task and environment). The third layer corresponds to behavioral outcome - the observable part of the improvisation process at the behavioral level - which can be easily captured by means of movement analysis and made readable using dimension reduction techniques (e.g., multidimensional scaling to display the individual motor signatures). In this way, each individual’s trial was plotted as a point on a map, so that similar trials are placed near each other and dissimilar trials are placed far from each other. The area of ellipses that encompasses all dots (experimental trials) of each individual provides a measure of within-person motor (intra-individual) variability (i.e., creativity landscape explored).

There is now growing evidence that improvisers dynamically act and react according to a set of changing constraints (e.g., Newell, 1986), including individual constraints (e.g., level of expertise), task constraints (e.g., rules: improvising on a given theme) and environmental constraints (e.g., the presence of an audience). With this in mind, one can thus consider improvisation activity as an on-going dynamic process involving a search for adaptive and creative (motor) solutions to a variety of constraints. We argue that creative solutions that best fit the situation emerge through exploration, under the interacting constraints imposed onto the improviser (Orth et al., 2017), and that motor variability is a key component for improvisation expression. Figure 1 illustrates our claim.

Each individual is assumed to have a more or less broad (behavioral) repertoire reflecting the ontogenesis/history of the individual, including past experiences and level of expertise (see the first layer of Figure 1B). This behavioral repertoire is enriched over time and (sensorimotor) learning, and serves as support for improvisation on which it feeds. Thus, during improvisation performance, the improviser can exploit existing elements from his/her own repertoire or explore new combinations of these elements according to the requirements of the task and the environment. This is manifested at the behavioral level either as a routine pattern of motions or as new ways of moving (Torrents et al., 2010). Relevant to understanding where the inspiration underlying improvisation comes from, it is necessary to examine the nature of constraints, namely the interaction between the individual, his/her surrounding environment and the task demands (see the second layer of Figure 1B). Inspiration is fleeting. A theme, a story, an emotion, an object, or the interaction with a partner can trigger it. For instance, in football, dynamic interactions between players (teammate and opponent) can lead to creative solutions (e.g., offensive or defensive strategies) that cannot be explained solely by the actions of each individual (i.e., the whole is greater than the sum of individual actions; Sawyer and DeZutter, 2009). Other factors may more or less account for the emergence of certain improvised products. This is at least the case for memory, which is known to encode, store and retrieve information from past events (Baddeley, 1997), and influences future actions (Gärling et al., 1997). Specifically, research has demonstrated the crucial role of working memory in imagination and creativity capacities, enabling the construction of original materials using a fraction of all the information “stored” in participants’ memory, those recalled and manipulated at a specific instant of time (Ockelford, 2012). Time plays a major role since both memory and improvisation operate at different time scales, from a fraction of seconds (working memory/improvised motor act) up to a lifetime (long-term memory/expertise acquisition). Despite the fact that the working memory and the long-term memory operate at different time scales, some authors consider working memory as part of long-term memory and have introduced the notion of “long-term working memory” (Ericsson and Kintsch, 1995; Ericsson and Delaney, 1999). The idea behind this is that lifelong knowledge/skills/information acquired through learning/experiences and stored in long-term memory are kept directly accessible by means of retrieval cues in working memory (Ericsson and Kintsch, 1995). Given the spontaneous character of improvisation performance, we believe that the temporal constraint significantly shapes improvised behaviors. The generation of behavior on a fast time scale would be mainly determined by previous training and learned automatisms, and is therefore likely to be “less” original than the generation of behavior on a slower time scale. Exploration phases of new improvised behaviors indeed require much longer time than the exploitation of existing improvised behaviors. We also believe that social interactions, as part of environmental constraints, significantly shape our repertoire and constitute a real catalyst for the discovery of novel behaviors. This is based on a large body of evidence suggesting that when we move with other people, the overlap between action observation and action execution is responsible for a process of motor interference (or contagion) that arises during both transitive (goal-oriented actions; Hardwick and Edwards, 2011) and intransitive (non-goal-directed actions; Kilner et al., 2003) actions. In joint improvisation tasks, since individuality challenges interpersonal motor coordination, individuals must partially or totally lose their individual preferences to behave similarly to their partner at the time of interaction, therefore expressing a certain type of behavioral plasticity. Słowiński et al. (2016) and Hart et al. (2014) reported substantial changes in individuals’ motor behaviors (signatures) upon social contacts (environmental change). Such behavioral changes induced by social interactions may persist even after the encounter is over, the so-called social memory effect (Oullier et al., 2008; Nordham et al., 2018). However, differences can be expected depending on the purpose of the task (spontaneous or intentional motor coordination). In the case of spontaneous settings, individuals are “freer,” so they can create more complex and unusual motion patterns without worrying about (the coordination with) their partner. However, they can lose the potent beneficial effect of entrainment on social memory, as the quality of the information exchanged during spontaneous interaction is degraded compared to that of intentional coordination. On the other hand, intentional coordination often suffers from a lower creativity level (Issartel et al., 2017) in order to produce simple patterns of movement that are easy to predict (Hart et al., 2014), but social memory may nevertheless benefit from the effect of entrainment. Future studies can potentially address these issues by investigating the effect of constraints on improvisation capacities.

## Moving Forward

In this Perspective article, we emphasized that improvisation skills are not purely cognitive products but are shaped and expressed through body motion. Extracting and analyzing the continuous stream of information offered by movements during a performance opens new promising avenues for a better understanding of the creative and learning processes involved in improvisation.

Our proposal for studying improvisation can be summarized as follows:

(i) *Capturing the essence of improvisation in motor performance in the most naturalistic and reliable way as possible*. The *mirror game* (*MG*) seems to be a good model (see Noy et al., 2011; Feniger-Schaal and Lotan, 2017 for full body 3D mirror game) because it provides a good balance between naturalistic (social) interaction and controlled interaction inspired by one of the most common drama exercises. In addition, *MG* entails all the ingredients to provide access to the motor expression of creative processes. That is, it enables exploratory behavior in which participants play together and search for various interesting patterns of movement by varying both amplitude and frequency of motions during the trial. Movement patterns during *MG* contrast thus with the simple rhythmic movements (mono-frequency and fixed amplitude) traditionally obtained in coordination studies with very restrictive motor tasks such as pendulum oscillation (e.g., Coey et al., 2011), finger tapping (e.g., Nowicki et al., 2013) and rocking-chair (e.g., Richardson et al., 2007). *MG* has however some limitations for the study of improvisational skills. First, *MG* might primarily be an exercise in coordination rather than improvisation, with interpersonal coordination playing a major role in joint improvisation. We therefore suggest that *MG* can be employed as a model for studying the effect of coordination on joint improvisation performance (dyad) or collective performance (group more than two players – Alderisio et al., 2017). As a precaution, solo performance should be evaluated first, as well as just after the interaction, to assess the effect of interpersonal coordination on individual behaviors and the persistence of these changes over time. A second limitation of *MG* is that the original experimental (one-dimensional) set-up is oversimplified, and therefore may not account for non-trivial daily activities such as driving a car or talking to another person, which are all more or less improvised from both motor and cognitive points of view. Moreover, it does not allow to investigate properly solo improvisation skills or the aesthetic quality of the performance. Consequently, further investigations in more ecological solo and collective improvisation tasks seem particularly relevant. To this end, researchers can rely on recent technological advances in 3D motion capture (e.g., marker-less tracking) and in (big) data analysis with artificial intelligence algorithms to better understand both individual and collective improvisation across various contexts (artistic, sports, as well as daily).
(ii) *Using relevant metrics to analyze kinematic data to infer the underlying creative processes involved in the improvisation performance and to identify key features of expertise*. We suggest that the extraction of behavioral signatures (e.g., Słowiński et al., 2016) can help us to discriminate creativity in participants and relate it to their levels of improvisation expertise. For example, Słowiński et al. (2016) proposed an index able to capture the subtle differences in the way each person moves in *MG*. Both intra- (between trials) and inter-personal (between players) motor variability was used as input to demonstrate that the individual motor signature of a person is time-invariant and that it significantly differs from those of other individuals. Interestingly, the authors noticed that some participants had a quite specific way of moving that was preserved over time (i.e., across trials) while others tended to change their movement between trials. In light of the instructions given to the participants—“*Play the game on your own, create interesting motions and enjoy playing*”—this intra-individual motor variability seems to somehow reflect participants’ creativity. We strongly believe that motor variability might be a reliable marker of creativity. The underlying assumption is that creative motor solutions arise – in some manner – from the continuous stream of variations in motor acts. The larger the variability, the more likely it corresponds to a (statically rare) creative motor solution (Orth et al., 2017). A deeper analysis of improvisation skills can be done by measuring the level of performance, particularly in the achievement of the final goal of the task. In the case of an artistic piece, performance can be evaluated, in a non-exhaustive way, by its behavioral richness in line with the theme, the originality of created actions or its aesthetic value (perceptual judgments). With regard to conversation, the purpose of the task is slightly different, and other performance markers should be used (e.g., quality of exchanges and information retained; interaction time; psychological factors such as affiliation, feeling of connectedness or interpersonal rapport). All these performance markers must thus be taken into account in the definition of motor signatures by correlating movement features to be extracted with these markers.
(iii) *Playing with the set of constraints* (*individual, task, environmental*) *to directly test their effects on behaviors and learning*. We suggest juggling the various constraints to assess the extent to which improvisational skills are domain-specific or domain-general. For instance, by analyzing an individual in solo and duo situations to evaluate the effect of social environment or by changing the type of the task. Longitudinal studies in a modified or unchanged environment, still lacking in the literature, can also be conducted to investigate more deeply the learning process and identify the various stages underlying the construction of improvisation skills.

## Author Contributions

AC conceptualized the perspective piece and wrote the first draft of the manuscript. BB and LM revised the manuscript and supervised the whole process. All authors approved the final version of the manuscript.

## Conflict of Interest

The authors declare that the research was conducted in the absence of any commercial or financial relationships that could be construed as a potential conflict of interest.
